# n-Si/p-NbSe_2_ Heterojunctions Designed as Color-Selective Photodetectors for Visible-Light Communication

**DOI:** 10.3390/s26123939

**Published:** 2026-06-21

**Authors:** Seham R. Alharbi, Atef F. Qasrawi, Laila H. Gaabour

**Affiliations:** 1 Department of Physical Sciences, Faculty of Science, University of Jeddah, Jeddah 23442, Saudi Arabia; 2 Department of Physics, Arab American University, Jenin P298, Palestine; atef.qasrawi@aaup.edu; 3 Department of Electrical and Electronics Engineering, Istinye University, 34010 Istanbul, Turkey

**Keywords:** n-Si/p-NbSe_2_ visible light communication, rectifiers, responsivity, detectivity

## Abstract

Herein, p-NbSe_2_ thin films were deposited onto n-Si substrates to fabricate an n-Si/p-NbSe_2_ (SNS) heterojunction for visible light communication (VLC) applications. Structural analysis revealed that the NbSe_2_ films possess a trigonal phase and are composed of slightly elongated and irregularly shaped grains with an average size of 0.131 μm. Electrical characterization showed that the SNS heterojunction exhibits pronounced rectifying behavior, with a bias-dependent asymmetry factor reaching 6.6 × $10^{3}$. The photodetection performance of the device was evaluated under illumination from white, blue, red, tungsten, and infrared LEDs. The device exhibited excellent photodetection characteristics across the visible region, achieving a maximum responsivity of 3.79/3.68 AW^−1^, external quantum efficiency of 1160/809%, noise equivalent power of 4.43 × 10^−14^ /4.57 × 10^−14^ WHz^−1/2^, and specific detectivity of 3.91 × 10^12^/3.79 × 10^12^ Jones under blue/white light illumination, confirming its practical relevance for VLC systems. In addition, frequency-dependent photocurrent measurements under modulated blue and white LED illumination revealed −3 dB bandwidths of approximately 775 Hz and 716 Hz, respectively, supporting the potential of the n-Si/p-NbSe_2_ photodiode for low-frequency VLC-related visible-light detection. Compared with previously reported photodiodes used in VLC and IR technologies, the present device demonstrated superior responsivity and EQE%, together with competitive NEP and detectivity. The enhanced performance is attributed to efficient photocarrier generation and collection across the Si/NbSe2 heterojunction. These results confirm that the fabricated SNS photodiode is a promising candidate for high-sensitivity and efficient visible light communication applications.

## 1. Introduction

VLC technology has become attractive as a wireless communication platform due to its use of the unregulated visible spectrum, security, minimal electromagnetic interference, and compatibility with solid-state illumination for various purposes, including networking, automotive communication, underwater data transfer, and sensing applications. Still, there remain challenges in the development of practical VLC devices that need to possess a fast response, visibility, low noise, ease of fabrication, and low cost. This is what continues to drive research into the discovery of novel heterojunction materials other than the traditional detector devices [1].

In recent years, the interest in using transition metal dichalcogenides, such as NbSe_2_, for optoelectronic applications has grown due to their high conductance, advantageous carrier transport properties, mechanical flexibility, and suitability for van der Waals heterostructures. The use of NbSe_2_ for optoelectronic applications appears to be effective, where NbSe_2_ serves as a carrier transport or carrier collection layer. Examples include UV-visible photodetectors based on the NbSe_2_/Nb_2_O_5_ heterostructure [2], self-powered Graphene/WSe_2_/NbSe_2_-based detectors [3] with a broad band response range suitable for optical communication and imaging applications. PdSe_2_/NbSe_2_ heterostructures have also been observed for polarization-selective photodetectors with broad spectral coverage [4].

Nevertheless, NbSe_2_ still faces several obstacles in terms of being effectively used in communication-related photodetectors. Firstly, due to its metallic nature, NbSe_2_ may result in relatively high dark currents if not embedded into the device structure using a junction field [5]. Secondly, many effective NbSe_2_ detectors require either exfoliation, dry transfer, or complicated van der Waals assembly processes that are not favorable from a scalability perspective [5]. Lastly, although NbSe_2_-based heterostructures are known to work well at both broadband and optical communication-related ranges, there is almost no research aimed at creating devices based on NbSe_2_ and silicon for simple structures operating with VLC-related wavelengths [6].

One possible approach towards solving such challenges would be utilizing silicon and NbSe_2_-based heterojunctions. To begin with, silicon still appears to be one of the most common materials used for visible-range detection due to its inexpensive and mature processing techniques [7,8,9]. Moreover, by creating the structure of n-Si/p-NbSe_2_, one can create a built-in junction ensuring an efficient separation of the photo-generated carriers and, thus, the use of transport properties of NbSe_2_ and absorption features of silicon simultaneously. At the same time, thermal evaporation can be used to make the process even simpler. These motivations may also be extended to advanced optical communication links, including satellite-assisted and deep-space optical communication, where efficient and low-noise photodetectors remain highly desirable, as recently discussed by Gao et al. [7].

With regard to the above motivations, we demonstrate, in this work, the fabrication of an n-Si/NbSe_2_ (SNS) heterojunction photodiode using the thermal evaporation method and investigate its electrical properties under the conditions of dark, blue, white, red, tungsten, and infrared light. Our research is centered on the detection characteristics that are crucial in determining the quality of the photodiode, such as the detector responsivity, external quantum efficiency, noise-equivalent power, and specific detectivity.

## 2. Experimental Details

Commercial plasma-etched n-type silicon wafers with a thickness of 300 μm were used as a substrate in this work. The NbSe_2_ thin films were deposited by the thermal evaporation technique using a VCM-600 thermal evaporator (Athena, Greece) onto these substrates. During deposition, the chamber pressure was maintained at approximately 10^−5^ mbar. Before the deposition process, the substrates were chemically cleaned to remove impurities and to ensure good film adhesion and interface quality. The substrates were placed at a distance of 22 cm above the evaporation source. After deposition, Ag electrodes (99.999%, Alfa Aesar, Ward Hill, MA, USA) of rectangular shape were formed on the film surface, while an additional electrode was deposited on the opposite side of the n-Si wafer to complete the n-Si/NbSe_2_ (SNS) heterojunction photodiode structure. The 1.0 μm thickness of the deposited films was determined using an Inficon STM-2 high-resolution thickness monitor. The crystal structure of the prepared layers was analyzed by X-ray diffraction (XRD) using a Rigaku Miniflex 600 diffractometer. The surface morphology was examined using a COXEM 200 scanning electron microscope (SEM) (COXEM Co., Ltd., Daejeon, Republic of Korea). The composition was determined using an X-ray fluorescence unit. The electrical characteristics of the fabricated SNS heterojunction were studied through current-voltage (I-V) measurements using a Keithley I-V system (Solon, OH, USA) under dark and illuminated conditions. The photoresponse behavior of the device was examined under different illumination sources, including blue, white, red, tungsten, and infrared light. The photocurrent spectra and optical transmittance and reflectance were recorded with the help of a thermo-scientific Evolution 300 spectrophotometer (Waltham, MA, USA). The low-frequency dependence of the photocurrent was recorded using a signal generator (1.0–5.0 MHz) to tune the diode pulses.

## 3. Results and Discussion

### 3.1. Structure, Composition, and Morphology

**Figure 1:**
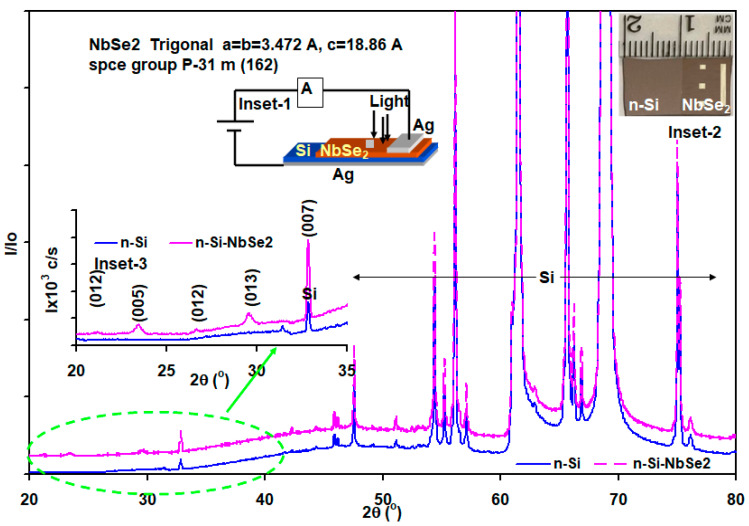
The XRD patterns for n-Si wafers and for trigonal NbSe_2_ films coated onto n-Si wafers. Inset-1 and Inset-2 showing the schematics of the device, while Inset-3 represents an enlargement of the less intensive diffraction patterns in the low diffraction angle region.

The study here focuses on reporting the basic properties and applications of NbSe_2_ thin films deposited onto n-Si wafers and their applications as photodiodes suitable for visible light communication (VLC) technology. The schematic and optical images for the proposed devices are shown in Inset-1 and Inset-2 of Figure 1. The optical image showed a slight difference in the colors of the film as compared to the substrate, whilst the schematic additionally demonstrates the circuit diagram of the device channels. Figure 1 displays the X-ray diffraction (XRD) patterns for the device under study. The figure includes the XRD patterns of the Si wafer as compared to the n-Si/p-NbSe_2_ (abbreviated as SNS) bilayers. The XRD patterns of the epilayer (NbSe_2_) are also enlarged in Inset-3 of the same figure. Deep analyses using “CrystDiff version 6.1” software packages and the literature data [10] showed that the observed sharp peaks are all assigned to the trigonal phase of NbSe_2_ and to cubic Si (*a* = 5.441 Å, JCPDS card No. 34–0394). Trigonal NbSe_2_ exhibits lattice parameters of *a* = *b* = 3.472 Å and *c* = 18.86 Å. The space group of this system is −31 m. Trigonal NbSe_2_ is formed by stacked Se-Nb-Se layers, in which each Nb atom is coordinated by six Se atoms in a trigonal-prismatic arrangement. In the common 2H-NbSe_2_ polytype, these Se-Nb-Se sandwiches are held together by weak van der Waals forces [10].

On the other hand, the X-ray fluorescence (XRF) measurements have shown that the material is composed of 35.81% at. Nb and 64.19% at. Se, with atomic content ratios of Se/Nb of 1.79. The numerical data suggest that there is a Se deficiency or selenium vacancy. Se vacancies, which are defects and/or trap centers, can strongly affect the recombination centers. Se vacancies are mentioned, resulting in the conversion of shallow traps into recombination states that limit the effective minority carrier recombination time [11]. It also limits carrier extraction of the photovoltaic effect in devices [11]. Even though there is a Se vacancy, considering that the bonding between Si atoms with average bond lengths of 2.46 Å [12] being longer than that of Nb-Se (2.63 Å [13]) and Se-Se (2.29 Å [14]), the interaction between dangling atoms of Si with Se is preferable. In addition, as the ionic radius of Si^+4^ is 0.44 Å, which is less than that of Nb^+4^ (0.83 Å) [15], ionic substitution of Si^+4^ in sites of Nb^+4^ is preferable, leading to a stronger interaction between Si and Se at the ultrathin interface. Reducing dangling bonds in Si by interaction with NbSe_2_ decreases the amount of defects and trap centers at the interface [16].

**Figure 2:**
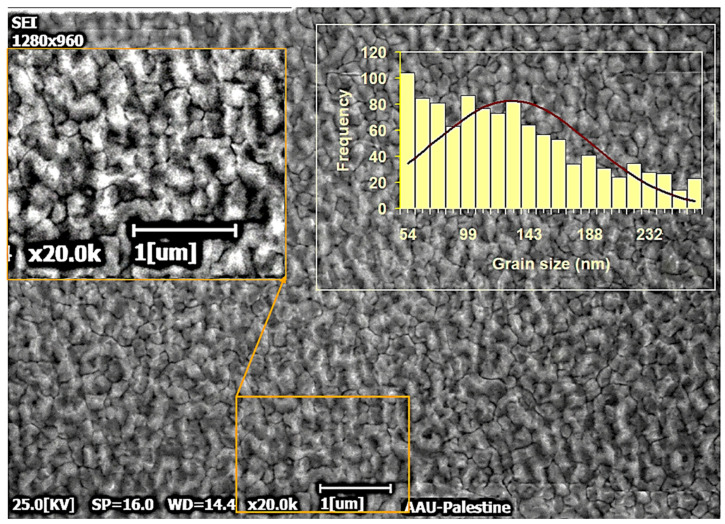
The scanning electron microscopy image for NbSe_2_ film deposited onto n-Si wafers, with the inset showing the histogram and the Gaussian distribution of the grain size analyses.

Figure 2 depicts the scanning electron microscopy (SEM) image of NbSe_2_ coated onto n-Si wafers. The SEM image reveals the existence of highly dense and relatively homogeneous fine-grain surface morphology characterized by grains that are equiaxial and slightly elongated and irregular in shape, forming a microstructure that is compact without any noticeable large pores and cracks within the examined area. The grain sizes were determined based on the SEM image through image analysis with calibration from the 1 μm scale resulted in an average grain size of about 0.131 um with a standard deviation of 0.058 um. The respective grain size distribution is shown in a histogram (inset of Figure 2) with the grain size plotted against the frequency, where a Gaussian plot is also plotted to provide an idea about the distribution trend. The measured distribution indicates a narrow spread of ultrafine grains, consistent with a fairly uniform microstructure within the analyzed field. Notably, the software analyses related to the SEM image included 1083 estimated grain counts with the dominant equiaxed to slightly elongate grains.

### 3.2. Optical Absorption and Spectral Photoresponse

**Figure 3:**
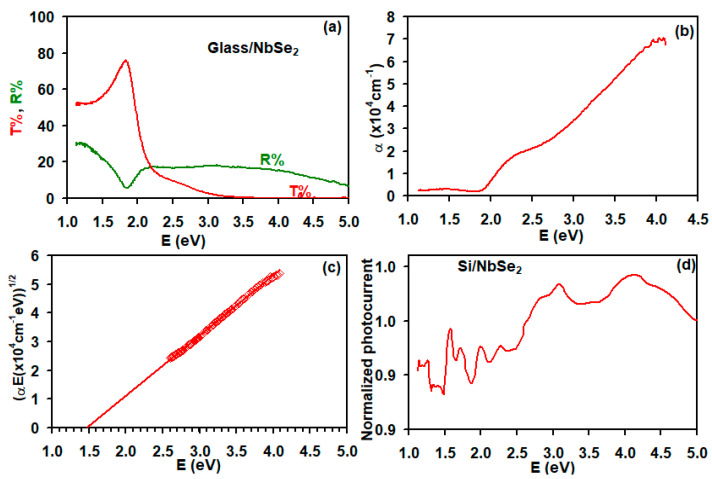
Optical properties and spectra of the NbSe_2_ thin film and n-Si/p-NbSe_2_ heterojunction: (**a**) optical transmission and reflection spectra of glass/NbSe_2_, (**b**) absorption coefficient versus photon energy spectrum, (**c**) Tauc plot used for determination of optical band gap, and (**d**) spectral response of n-Si/p-NbSe_2_ photodetector.

The optical properties of the NbSe_2_ thin film and the photodetection spectra of the fabricated n-Si/p-NbSe_2_ photodetector are provided in Figure 3. From Figure 3a, the glass/NbSe_2_ film shows considerable transmittance (T%) within the low photon energy region, while there is a steep drop-off in transmittance in the vicinity of 1.8–2.0 eV photon energy. As such, it is evident that the strong optical absorption occurs within the visible photon region. The reflectance (R%) spectra for the glass substrate/NbSe_2_ thin film are moderate and do not vary significantly within the test photon energy range. As such, it can be concluded that the variation in transmittance is due to photon absorption in the NbSe_2_ thin film. The absorption coefficient (α) values for the fabricated film were determined from the equations, $T=(1-R_{glass})(1-R_{film})e^{-\alpha d}$ [17], where $R_{glass}$ and $R_{film}$ refer to the contribution of the glass substrate and NbSe_2_ thin film to the reflected signal, and *d* refers to the thin film thickness. The results obtained are shown in Figure 3b. The obtained α values increase with photon energy, confirming enhanced absorption toward the blue/near-UV region. On the other hand, from the Tauc plot shown in Figure 3c, the direct optical band gap was calculated using the relationship of ${\left(\alpha E\right)}^{1/2}\propto \left(E-E_{g}\right)$ [17], with E being the energy of incident photons. The estimated direct optical band gap for the NbSe_2_ film is about 1.5 eV. This optical band gap value is similar to that of NbSe_2_ nanoparticles at about 1.42 eV, implying that the present sample film has a proper absorption edge suitable for visible and near infrared light photodetection [18]. Moreover, it should be highlighted from Figure 3d that the spectral photoresponse of the heterojunction of n-Si/p-NbSe_2_ is extensive within the entire visible spectrum. Importantly, an enhanced response towards high-energy photons corresponding to the blue spectrum is observed. This enhanced response towards the blue region is significant as it implies that the heterojunction can efficiently detect the blue component in the visible spectrum. Since the blue region excitation in LEDs has been widely used directly in the VLC technique, the wide response of the heterojunction will imply its effective detection of blue light emission and operation.

### 3.3. SNS Photodiode Characteristics

In Figure 3c, we have shown that NbSe_2_ films exhibit an indirect transition optical gap of 1.50 eV. The value is also reported elsewhere as 1.42 eV and 1.50 eV [18]. Although the extracted optical gap of 1.50 eV appears unusual for ideal crystalline 2H-NbSe_2_, which is generally regarded as metallic, this value should not be interpreted as a fundamental band gap of pristine NbSe_2_. The proposed SNS photodiodes are composed of Ag/n-Si/p-NbSe_2_/Ag. The bottom Schottky electrode at the Ag/ n-Si is accompanied with a barrier of height of (${q\phi }_{1}=\left|q\chi_{n-Si}-{q\phi }_{Ag}\right|=\left|4.05-4.73\;eV\right|=$ 0.68 eV, whilst the top Schottky devices at the Ag/NbSe_2_ interface, reveal a barrier height of (${q\phi }_{2}=\left|q\chi_{p-NbSe2}+E_{g}-{q\phi }_{Ag}\right|=\left|4.41+1.50-4.73\right|=1.18$ eV. The former are barriers to the motion of electrons, whilst the latter is a barrier for holes. The conduction band offset at the n-Si/p-NbSe_2_ interface is, $\Delta E_{c}=\left|{q\chi }_{n-Si}-{q\chi }_{NbSe2}\right|=\left|4.05-4.41\right|$ = 0.36 eV and valence band offset is $\Delta E_{v}=\left|\Delta E_{g}-{\Delta E}_{c}\right|=\left|0.30-0.36\right|$ = 0.06 eV. For the SNS device, a large conduction-band offset (${\Delta E}_{c}$ = 0.36 eV) means electron transfer is hindered at the n-Si/p-NbSe_2_ interface, while the very small valence-band offset (${\Delta E}_{v}$ = 0.06 eV) indicates holes can transfer much more easily. Thus, the junction is expected to be hole-selective, promoting hole extraction and suppressing electron back-transfer/interfacial recombination. However, as mentioned above, the deposition of Ag on both sides leads to the formation of Schottky contacts at Ag/n-Si and Ag/p-NbSe_2_, with barrier heights of 0.68 eV and 1.18 eV, respectively. Together with the band offsets at the n-Si/p-NbSe_2_ heterointerface (${\Delta E}_{c}=$ 0.36 eV and ${\Delta E}_{v}=$ 0.06 eV), these barriers determine the carrier transport across the device. The larger conduction band offset and the Ag/n-Si Schottky barrier suppress electron transfer, while the very small valence band offset facilitates hole transport across the heterojunction. Meanwhile, the high barrier at the Ag/p-NbSe_2_ interface imposes stronger resistance to carrier collection, indicating that this contact is the dominant blocking barrier in the structure [19]. In practice, under forward bias from the n-Si side, the junction barrier is lowered, promoting carrier transport across the n-Si/p-NbSe_2_ interface. Owing to the small $\Delta E_{v}$, hole transport is favored, while electron transport is hindered by the larger ${\Delta E}_{c}$. Although the Ag/n-Si contact has a moderate Schottky barrier (0.68 eV), the much higher barrier at Ag/p-NbSe_2_ (1.18 eV) remains the dominant obstacle, making this interface the main current-limiting contact under forward bias.

It should be noted that the proposed band alignment of the n-Si/p-NbSe_2_ heterojunction was constructed using reported electron affinity/work-function values and the optical band gap obtained from the present absorption analysis. This approach provides a reasonable first-order estimation of the interface energetics; however, it should be noted that the exact band offsets can be affected by interface states, native oxide, surface dipoles, defects, and Fermi-level pinning. In the literature, experimental techniques such as ultraviolet photoelectron spectroscopy (UPS), X-ray photoelectron spectroscopy (XPS), valence-band analysis, Kelvin probe measurements, and scanning tunneling spectroscopy have been widely used to directly determine work functions and valence/conduction band offsets in semiconductors and two-dimensional-material heterostructures. For example, Chiu et al. determined the band offsets of a MoS_2/_WSe_2_ heterojunction using microbeam XPS and scanning tunneling microscopy/spectroscopy, demonstrating the importance of direct experimental band-alignment characterization in layered heterostructures [20]. Therefore, the band diagram presented here should be regarded as a literature-assisted estimated model that supports the observed rectifying behavior and photoresponse, while direct UPS/XPS/Kelvin probe measurements are recommended as future work for quantitative validation of the n-Si/p-NbSe_2_ interface energetics.

**Figure 4:**
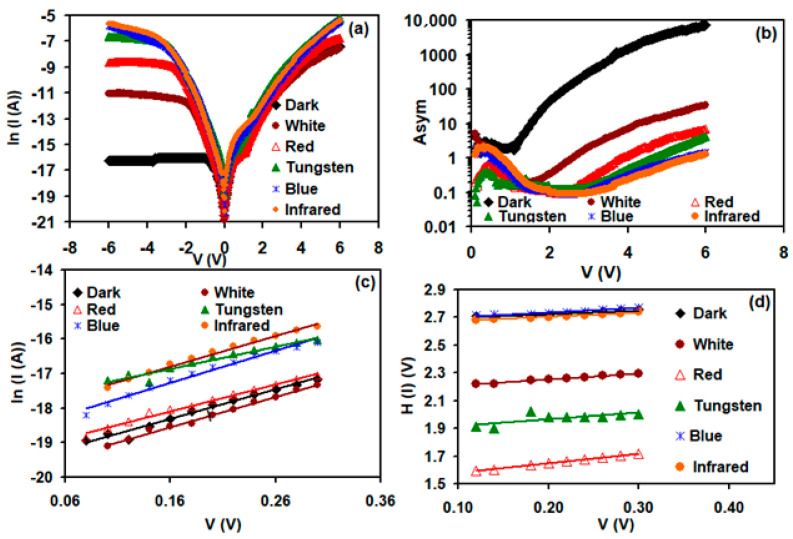
(**a**) The current-voltage characteristics curve, (**b**) the biasing-dependent asymmetry, (**c**) the Richardson–Schottky fitting for reverse current in the low reverse voltage range, and (**d**) the Chueng’s function for the SNS photodiodes.

To explore the SNS photodiode characteristics, the current (I)-voltage (V) characteristic curves were recorded at room temperature in the dark and under the illumination of light-emitting diodes (LED) of different colors, suiting visible light communication technology. The measured I−V curves are presented in Figure 4a. As seen from the figure, for all I−V curves, the forward current ($I_{F}$) is larger than the reverse current ($I_{R}$), indicating typical diode characteristics with rectification ratio or diode asymmetry ($Asym.=I_{F}/I_{R}$) being presented in Figure 4b. One can also see from Figure 4b that, for applied reverse biasing voltage less than $V_{c}=$ 1.0 V, Asym decreases with increasing applied voltage; this critical voltage ($V_{c}$) shifts toward larger voltages upon light excitation. $V_{c}$ is 1.28 V, 2.08 V, 2.36 V, 2.52 V, and 2.68 V, when the SNS device is excited with white, red, tungsten, blue, and infrared light, respectively. For all applied voltages larger than $V_{c},\;Asym$ increases systematically with increasing applied voltage, exhibiting a maximum value of 6.6 $\times \;10^{3}$ at 6.0 V. It can also be seen from Figure 4a that upon excitation, a leakage current dominates. When excited with white, red, tungsten, blue, and infrared lights for all $V_{R}>1.92$ V, 2.56 V, and 2.75 V, respectively, $I_{R}$ sharply increases with increasing voltage. Such an increase strongly lowers the diode asymmetry. Particularly, as can be seen in Figure 3b, Asym increases with increasing voltage, reaching a maximum value of 6.6 $\times \;10^{3}$ at V=6.0 V in the dark and then decreasing, reaching a value of 31, 6.5, 3.4, and 1.2 upon excitation with white, red, tungsten, blue, and infrared lights, respectively. The data here indicate the ability to use the SNS devices as a rectifier and to control light excitations. Remembering that NbSe_2_ is a selenium-defective material, the domination of leakage current at high applied voltages can result from a trap-assisted tunneling of holes at the Schottky metal and from thermionic emission of electrons from the channel to the p-NbSe_2_ layer [21]. This means that beyond this bias, the current is no longer governed mainly by ideal thermionic carrier injection across the Ag/n-Si, n-Si/p-NbSe_2_, or Ag/p-NbSe_2_ barriers. Instead, additional non-ideal transport mechanisms start to dominate, such as defect-assisted tunneling, interface-state conduction, shunt leakage, or trap-assisted generation–recombination. In other words, at a higher bias, the blocking role of the junction/barriers weakens, and the current increasingly flows through leakage paths rather than the intended rectifying transport channel [22].

Figure 4a,b also demonstrate the light illumination effects on the I−V curves and on the biasing-dependent asymmetry. While the illumination effect is less significant under the forward biasing condition, the reverse current is highly affected by light illumination. Particularly, $I_{R}$ remarkably increased, leading to a noticeable decrease in the asymmetry values (Figure 4b). The increase in $I_{R}$ upon illumination by a few orders of magnitude indicates the remarkable impact of light on the “OFF” state of the heterojunction device. This enhanced behavior of $I_{R}$ is ascribed to the generation of additional electron-hole pairs, which increases the free carrier density, leading to improved current transport across the junction. In addition, as unavoidable approaches, the activation of trap centers and defects and interface states by light enhances the trap-assisted tunneling, leading to a further increase in $I_{R}$ [23].

It can be seen from Figure 4a that the response to tungsten, blue, and infrared light is more significant than to white and red lights. Physically, under reverse bias, the high photocurrent observed under 980 nm IR and tungsten, which also covers this region in the tungsten lamp, is consistent with the fact that its photon energy is about ~1.27 eV, which is still higher than the Si band gap (1.20 eV); therefore, efficient photogeneration can occur in the n-Si region. Silicon photodiodes are known to exhibit spectral sensitivity from the visible range up to the near-IR, with an upper cutoff near 1100 nm, which explains the strong response at 980 nm [22]. The similarly high current under tungsten illumination is attributed to the near-blackbody spectrum of tungsten lamps, which is particularly strong in the near-infrared region where Si remains highly responsive. In contrast, white and red illumination produce lower reverse current because carrier generation and collection depend strongly on wavelength-dependent absorption depth and recombination; short-wavelength blue light is absorbed near the surface, where recombination losses are more significant, while red light is absorbed more weakly than the shorter visible wavelengths. Consequently, the observed order IR ≈ tungsten > red is consistent with the spectral response of Si-based photodetection and the optical absorption profile of the heterostructure [24]. Apart from the role of n-Si for IR and tungsten lights, because their photon energy is lower than the reported NbSe_2_ band gap (1.5 eV), direct interband absorption in NbSe_2_ is expected to be weaker. However, NbSe_2_ still plays a crucial role by forming the p-side of the heterojunction, establishing the built-in electric field, and participating in the separation and transport of photogenerated carriers across the n-Si/p-NbSe_2_ interface. For this reason, the strong reverse photoresponse is not only governed by efficient light absorption in Si but also by the contribution of NbSe_2_ to the junction-assisted carrier extraction and interfacial charge transfer. The similarly high current under tungsten illumination is consistent with its strong emission in the red and near-IR region, where Si exhibits high responsivity [23,24]. The dependence of illuminated $I_{R}$ on the light energy means that the SNS photodiodes are promising for selective photodetection in visible-light communication (VLC) systems, where discrimination between different optical wavelengths is important for signal reception and channel separation [25].

On the other hand, relying on the Richardson–Schottky current ($I_{RS}$) transport mechanism, it was possible to determine how the ideality factor (n) and barrier height (${q\phi }_{b}$) are affected by the light energy. The Richardson–Schottky current is given by the relation [22,26,27]

(1) $$I_{RS}=(AA^{*}T^{2}e^{-\frac{q\phi_{b}}{kT}})(e^{\frac{qV}{nkT}}-1)$$

Here, the diode area $A=0.03\;{cm}^{2}$ for the square pad contacts (Inset-2 of Figure 1). $A^{*}=120m^{*}$ is the Richardson constant and the reduced effective mass $m^{*}={\left(m_{e-n-Si}^{-1}+m_{h-p-NbSe2}^{-1}\right)}^{-1}$ = ${\left(0.19^{-1}+0.96^{-1}\right)}^{-1}=0.158m_{o}$ [28]. As illustrated in Figure 4c, the linear fitting of the $\ln\left(I\right)-V$ in the low (0.08≤V≤0.30 V) and reverse biasing ranges in accordance with Equation (1) allowed us to determine the ideality factor and barrier heights in the dark and under illumination. The data is listed in Table 1. It is observed from the data that the ideality factor and barrier height depend upon the polarity of the bias and illumination conditions, indicating that carrier transport in the Ag/n-Si/NbSe_2_/Ag structure is influenced significantly by interfacial defects, barrier inhomogeneities, series resistance, and photogenerated carriers [22].

Table 1 also indicates that the SNS device exhibits strongly non-ideal transport under all conditions, since both $n_{F}$ and $n_{R}$ are much larger than unity, confirming the contribution of interface states, barrier inhomogeneity, recombination, high series resistance, and leakage current [22]. To reduce series resistance effects, a more professional approach known as Chueng’s functional analysis was employed. Chueng’s function ($H\left(I\right)$) is given by the relations [26]

(2) $$\frac{dV}{dln(I)}=\frac{nkT}{q}+IR_{s}$$

(3) $$H\left(I\right)=V-\frac{nkT}{q}ln\left(\frac{I}{AA^{*}T^{2}}\right)=n{q\phi }_{b}+IR_{s}$$

The fitting of the Cheung’s function for forward biased condition is shown in Figure 4d, whilst the results of the analyses for both of the forward and reverse biasing conditions are listed in Table 1. As seen from the figure, the fitting is linear, and the slope and intercepts allow us to determine the series resistance, ideality factor, and barrier height. The tabulated data indicate that in the dark, the series resistance for reverse biased ($R_{sR}$) condition is higher than the resistance of forward biased ($R_{sF}$) and sharply decreases upon illumination, displaying the lowest value of 0.27 MΩ for blue light followed by 0.42 MΩ for red and 0.55 MΩ for infrared light. Under illumination, the forward biasing condition showed less affected $R_{sF}$ values. The barrier height also significantly decreased under light due to the band bending at the junction [22]. Particularly, it is lowered because incident photons generate excess charge carriers that redistribute in the depletion region and at the interface, reducing the band bending and hence the effective barrier height [22]. In addition, the partial trap filling, where the interfaced and bulk traps capture charge carriers, alters the local electric field and shares in the reduction of band bending at the interface [27]. Although the values of the ideality factors (Table 1) are significantly reduced under light, their values are still larger than unity, indicating non-ideal transport such as recombination, interface states, and tunneling effects [22]. The changes in $n_{F}$ and $n_{R}$ with light type (wavelength) also show that the diode response depends not only on series resistance but also strongly on the incident light energy.

The larger changes under visible light illumination are consistent with stronger photoresponse, which, from the VLC point of view, means that the device can electrically distinguish different optical wavelengths. Such a property makes it a promising device for wavelength-selective optical detection [28].

### 3.4. Figures of Merit for SNS Photodiodes

In order to establish the photodiode parameters, the current responsivity (R), external quantum efficiency percentage (EQE%), noise equivalent power (NEP) and specific detectivity ($D^{*}$) were measured and calculated from the relations [22,29] as follows:

(4) $$R=\frac{I_{ph}}{P_{opt}}$$

(5) $$EQE\%=100\cdot \frac{Rhc}{e\lambda }$$

(6) $$NEP=\frac{\sqrt{2eI_{dark}}}{R}$$

(7) $$D^{*}=\frac{\sqrt{A}}{NEP}$$

Here, $P_{opt}$ is the optical power and λ is the light wavelength ($\lambda_{blue}=406\;nm,{\;\lambda }_{white}=565\;nm\;\left(averaged\right)$, $\lambda_{Red}=632.5\;nm$, $\lambda_{IR}=980\;nm$, and $\lambda_{Tungsten}=550\;nm\;(for\;visible\;region)$).

Recalling that the increase in the reverse voltage resulted in the onset of leakage current and that the light improved the reverse-bias carrier generation and extraction, the relation between the reverse biasing and photogeneration will identify the best operating range of the SNS photodiodes.

The diode figures of merit for SNS photodiodes under the tested light sources are listed in Table 2. In general, the devices show good current responsivities for all examined wavelengths. The highest current responsivity is recorded under blue light (3.79 AW^−1^), followed closely by white light (3.68 AW^−1^), then IR light (1.27 AW^−1^), red light (0.86 AW^−1^), and tungsten light (0.80 AW^−1^). The high responsivity under blue and white illumination indicates that the SNS photodiodes are suitable candidates for visible light communication (VLC) applications. In addition, the external quantum efficiency is highest for blue light (1160%), followed by white light (809%), then tungsten light (182%), red light (169%), and IR light (161%). *EQE*% represents how effectively incident photons are converted into collected charge carriers. Higher *EQE*% is associated with higher responsivity and improved photodetection performance. In general, higher *EQE*% values, especially for blue light (*EQE*% = 1160%), are associated with higher responsivity and improved photodetection performance. The *EQE*% value exceeding 100% under blue illumination indicates that the n-Si/p-NbSe_2_ photodiode operates with internal photoconductive gain rather than a simple one-photon/one-electron conversion mechanism. In the present device, this gain can be explained by the internal electric field at the n-Si/p-NbSe_2_ heterojunction, which promotes the efficient separation of photogenerated electron-hole pairs, together with charge trapping at the interface or defect states that extend the lifetime of one carrier type. If the carrier lifetime is longer than the carrier transit time, more charge carriers can be collected per absorbed photon, resulting in *EQE*% values higher than 100% [22]. Since no direct evidence of impact ionization, the avalanche multiplication factor, or excess-noise behavior was measured, the gain is assigned to a trap-assisted photoconductive mechanism rather than true avalanche multiplication. Moreover, the noise equivalent power (*NEP*) values are of the order of 10^−14^ WHz^−1/2^, indicating that very low optical power is sufficient to generate a signal equal to the noise level. The lowest NEP is observed for blue light (4.43 $\times \;10^{-14}$ WHz^−1/2^) followed by white light (4.57 $\times \;10^{-14}$ WHz^−1/2^), whereas the highest NEP is recorded for tungsten light (2.1 $\times \;10^{-13}$ WHz^−1/2^). Furthermore, the specific detectivity is also highest for blue light, with a value of 3.91 × 10^12^ Jones, followed by white light (3.79 × 10^12^ Jones), IR light (1.31 × 10^12^ Jones), red light (8.90 × 10^11^ Jones), and tungsten light (8.30 × 10^11^ Jones). These results confirm that SNS photodiodes exhibit their best overall performance under blue and white illumination, further supporting their suitability for visible light communication technology [9,29].

To address the noise contribution more accurately, the NEP and specific detectivity were recalculated by considering both shot noise and Johnson–Nyquist noise under the same reverse-bias condition used for the responsivity measurements. At −5 V, the reverse dark current was 88.1 nA, and the local dynamic resistance extracted from the dark I–V curve using $R_{d}=\frac{dV}{dI}$ was approximately 59.2 GΩ. The shot-noise current density was calculated as $i_{shot}=\sqrt{2qI_{dark}}=$1.68 × 10^−13^ A · Hz^−1/2^, while the Johnson–Nyquist noise current density was estimated using $i_{J}$ = $\sqrt{4kT/R_{d}}$ = 5.29 × 10^−15^ A · Hz^−1/2^ at room temperature [30]. The total estimated white-noise current density was then obtained from $i_{n}=\sqrt{i_{shot}^{2}+i_{j}^{2}}$ and used to calculate $NEP=\frac{i_{n}}{R}$ and $D^{*}=\sqrt{A}/NEP$. Using an active area of 0.03 cm^2^, the corrected estimated NEP values were 4.43 × $10^{-14}$ and 4.57 × $10^{-14}$ W Hz^−1/2^ for blue and white illumination, respectively, while the corresponding $D^{*}$ values were 3.91 × $10^{12}$ and 3.79 $\times \;10^{12}$ Jones. These values should be regarded as estimated white-noise-limited parameters, since low-frequency 1/f noise was not directly measured and may require future noise spectral density measurements.

**Figure 5:**
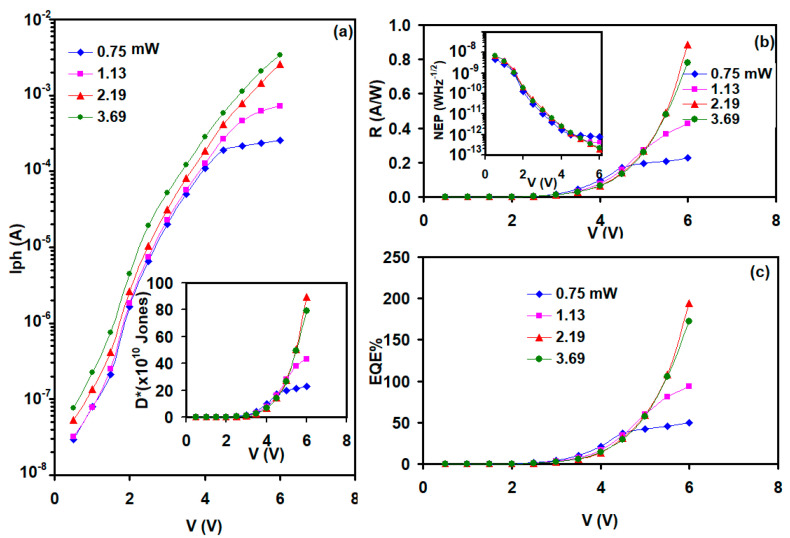
The reverse biasing effects on (**a**) the photocurrent, (**b**) current responsivity, and (**c**) external quantum efficiency. Inset of (**a**), and inset of (**b**) show the reverse biasing effects on the specific detectivity and noise equivalent power, respectively.

Figure 5 shows the effect of reverse biasing voltage (electric field effect) on the photocurrent for light irradiated from a mini tungsten lamp. It can be seen from the figure that the photocurrent increases sharply with increasing $V_{R}$ and increasing light power. For example, for the light power of 1.13 mW, increasing the biasing voltage from 1.0 V to 6.0 V increased the photocurrent from 32 nA to 0.70 mA, respectively. In addition, at a constant biasing voltage of 6.0 V, the photocurrent increases from 0.25 mA to 3.4 mA as the light power increases from 0.75 mW to 3.69 mW, respectively. On the other hand, as seen from Figure 5b, Figure 5c, and the inset in Figure 5a, the current responsivity, EQE% and $D^{*}$ increased with increasing reverse biasing voltage and increasing light power. The noise equivalent ratio displayed in the inset of Figure 5b decreased with increasing biasing voltage and increased with increasing light power. Actually, an increase in reverse-bias voltage enhances the photodiode’s carrier-collection efficiency by widening the depletion region and increasing the internal electric field [22,29]. As a result, photogenerated electron-hole pairs are separated and swept to the contacts more rapidly, which reduces recombination losses and increases the photocurrent. Since current responsivity is given by Equation (4), any bias-induced increase in the photocurrent at the constant incident optical power leads to a corresponding increase in responsivity. In addition, if the avalanche effect were to dominate under reverse biasing conditions, it would cause a large multiplication of the photocurrent because the high reverse electric field accelerates the photogenerated carriers to very high energies, and those carriers then collide with the lattice atoms strongly enough to create additional electron-hole pairs by impact ionization. These newly created carriers are also accelerated and generate even more pairs, so the current gets multiplied [22,29]. However, the measured Se/Nb atomic ratio of 1.79 indicates a Se-deficient NbSe_2_ film, suggesting the presence of Se-vacancy-related defect states. These defects are expected to play an important role in the electrical transport and photodetection behavior of the n-Si/p-NbSe_2_ heterojunction. In the proposed band model, Se vacancies introduce localized trap states within the NbSe_2_ band structure and near the n-Si/p-NbSe_2_ interface. Under dark bias, these states can act as intermediate levels for trap-assisted tunneling and recombination, which explains the non-ideal diode behavior, high ideality factors (Table 1), and leakage current observed in the device. In addition, defect scattering and imperfect interface formation can contribute to the MΩ-range series resistance. Under illumination, the built-in electric field at the heterojunction separates photogenerated electron-hole pairs, while Se-vacancy/interface trap states can temporarily capture one carrier type and prolong its lifetime. When the carrier lifetime becomes longer than the carrier transit time, multiple carriers can be collected per absorbed photon, giving rise to internal photoconductive gain and EQE% values above 100%. Therefore, the Se deficiency is not treated as an isolated compositional deviation but as a central factor governing the trap-assisted transport, non-ideal junction behavior, and gain-assisted photodetection mechanism of the n-Si/p-NbSe_2_ photodiode.

In the same context, recent reports continue to establish that defect-related phenomena, dimensional manipulation, and charge transport at interfaces are some crucial parameters in governing the functionality of nanoscale optoelectronic and sensing systems. For instance, light-weighted three-dimensional nanotubes of TiO_2_ grown on titanium mesh exhibited increased photocurrent through improved light capture, increased active surface area, and effective charge transportation channels [31]. Besides, NiSe_2_ has emerged as one of the two-dimensional material-based saturable absorbers for broad-spectrum photonic functionalities [32]. Moreover, recent research has revealed that WO_3_ functionalized V_2_CT_x_ nanosheets are effective room temperature wireless sensors of NO_2_ gas owing to effective tuning of surface/interface characteristics of two-dimensional MXenes [33]. The results provide evidence of the fact that non-stoichiometry, vacancy defects, interface effects, surface chemistry, and other properties are crucial in controlling carrier transport, recombination, trapping, and photoconducting activity of the semiconductor material. As such, in the present case of the n-Si/p-NbSe_2_ heterojunction, the deficiency of Se atoms plays an important role in defect-related charge carrier transport and gain mechanisms.

Table 3 compares the figures of the merit of the developed n-Si/p-NbSe_2_ SNS photodiode with those of other photodiodes utilized in VLC and IR applications. The current device possesses the highest value of responsivity (3.68 AW^−1^) compared to the respective figures of merit of 0.12 AW^−1^, 1.07 AW^−1^, 0.44 AW^−1^, 0.44 AW^−1^, and 0.51 AW^−1^ of other photodiodes of Refs. [34,35,36,37,38]. Additionally, the external quantum efficiency of the developed device is higher (809%) as compared to the values ranging from 15% to 125% of the literature devices. Thus, it is clear from the above-mentioned parameters that the SNS photodiode demonstrates enhanced photoresponse and conversion efficiency from photons to carriers. The noise equivalent power value is 4.57 $\times \;10^{-14}$ W Hz^−1/2^ in the case of the developed photodiode, which is lower than the respective figures of merit of polyoxometalate/p-Si [35] and those of Mg_2_Si/Si [36] and Nb_2_O_5_/n-Si [37]. Moreover, the specific detectivity is 3.79 $\times \;10^{12}$ Jones, which is higher than those of other photodiodes. Therefore, the present work provided an optimized design of a photodiode suitable for VLC applications.

It must be understood that the comparisons made in Table 3 have been made qualitatively and not necessarily as an absolutely normalized performance measurement of devices. This is due to the fact that while the literature devices mentioned in Table 3 were fabricated by applying different methods, the devices work at different illumination wavelengths, optical powers, and power densities, with different active areas and other parameters not specified or unavailable from their corresponding references. Thus, although incomplete in its normalization process, the device can still be qualitatively evaluated on its performance against previously published photodetectors for VLCs and IRs.

**Figure 6:**
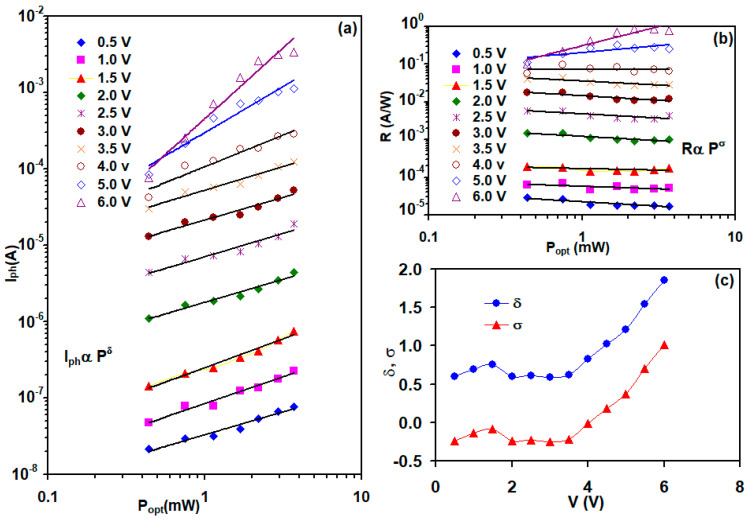
The light power dependence of (**a**) the photocurrent and (**b**) current responsivity for SNS visible light photodetectors, recorded as a function of electric field. (**c**) Shows the applied reverse voltage effect on the power exponents of photocurrent and responsivity, respectively.

On the other hand, the light power effect on the photocurrent and responsivity of the SNS photodiodes being recorded at various reverse biasing voltages are presented in Figure 6a and Figure 6b, respectively. Figure 6a show that the photocurrent increases with increasing light power. The $I_{ph}-P_{opt}$ dependence is of logarithmic type ($I_{ph}\propto P_{opt}^{\delta }$) exhibiting power exponent (δ) values that depend on the biasing voltage. Moreover, δ values highly depend on the applied voltage, and the relation between the power exponent δ and the applied biasing voltages are presented in Figure 6c. Low voltages showed δ<1.0 equation values, whilst reverse voltages higher than 4.5 V revealed δ>1.0 equation values. Consistent with this observation, the current responsivity (Figure 6b) decreased with increasing light power for all applied voltages less than 4.5 V. The relation between the current responsivity and illumination power is described by the equation, $R=P_{opt}^{\sigma }$. The power exponent σ which is calculated from the slopes of the $R-P_{opt}$ variations are presented as a function of reverse biasing voltage in Figure 6c. It is evident from the figure that σ is negative for all applied voltages less than 4.5 V. Below this value, the device performance is less effective at high light powers because the responsivity is weakened, whilst for all applied voltages larger than 4.5 V, the higher the light power is, the larger the current responsivity and the larger the generated photocurrent.

Physically, according to recombination theory, the dependence of photocurrent on optical power, $I_{ph}\propto P_{opt}^{\delta }$, provides insight into the dominant recombination pathway. A power exponent of 0.5 ≤ δ < 1.0 generally indicates trap-assisted recombination, whereas δ ≈ 1.0 corresponds to a nearly linear bulk-controlled response; values exceeding unity suggest a superlinear regime associated with enhanced carrier collection or gain at the interface/surface under higher excitation [39]. Therefore, the δ-V behavior shown in Figure 6c indicates that, to obtain high photoconductive gain, the reverse bias should exceed about 4.5 V, while below $V_{R}$ ≈ 4.0 V, the exponential trap distribution remains dominant and captures a significant fraction of the photogenerated carriers, leading to lower responsivity. This behavior can be reasonably related to selenium-vacancy-induced trap states in NbSe_2_, since Se vacancies are known to introduce defect states that strongly affect carrier transport and recombination in NbSe_2_ [40]. In parallel, the relatively improved response at higher bias may also reflect the role of a well-bonded Si–Se interfacial network at the Si/NbSe_2_ junction, which helps passivation of the Si dangling-bond-related interface states and facilitates more efficient carrier transfer across the hetero-interface [41].

**Figure 7:**
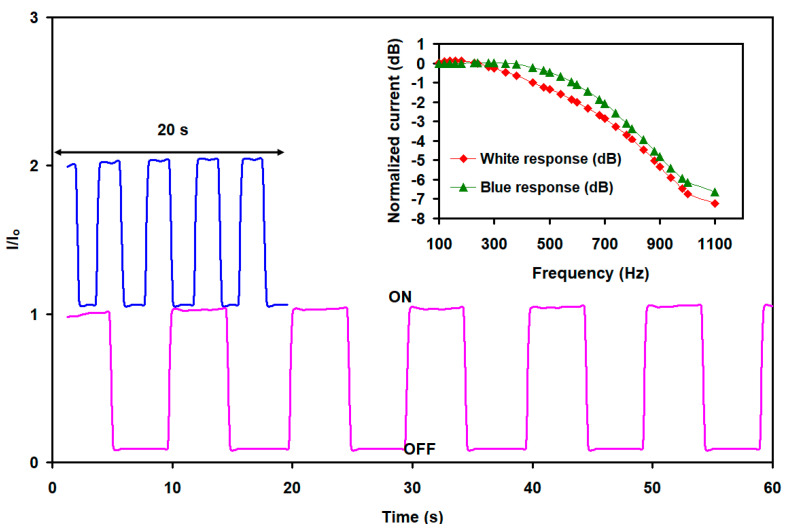
Dynamic and frequency-dependent photoresponse of the n-Si/p-NbSe_2_ photodiode under modulated visible illumination. The main panel shows reproducible ON/OFF switching behavior without prior electrical poling, while the inset presents the normalized photocurrent response in dB as a function of LED modulation frequency for white and blue illumination. The extracted −3 dB bandwidths are approximately 716 Hz and 775 Hz for white and blue LEDs, respectively.

Figure 7 demonstrates the switching dynamics and frequency dependence of the photoresponse in the n-Si/p-NbSe_2_ photodiode in a modulated visible light source. In particular, the ON/OFF time-dependent photocurrent transients reveal a reproducible photoresponse of the photodiode, suggesting the reversible generation and relaxation of the photocarriers within the heterojunction. Note that no electrical poling of the samples was performed prior to the measurements, demonstrating that the obtained data reflect only the intrinsic photoresponse properties of the investigated n-Si/p-NbSe_2_ structure and do not depend on the previously applied poling treatments. The rising and falling times of the photocurrents were determined based on the transient photocurrents by applying the standard 10–90% method. Namely, the rising time corresponds to the time interval at which the photocurrent increases from 10% to 90% of the maximum ON-state value, whereas the falling time corresponds to the time interval at which the photocurrent decreases from 90% to 10%. For further proof of the applicability of the photodetector in the field of visible-light communication, frequency-dependent photocurrent experiments were carried out on the modulation using blue and white LEDs in the range of 100–1100 Hz, as shown in the inset of Figure 7. At each modulation frequency, f, the photocurrent, $I_{ph}(f)$, was divided by the reference value ($I_{ph,\;0}$) obtained for $I_{ph}(f)$, in the 100–300 Hz range. This ratio was converted into a decibel form by defining Response (dB) = 20 log_10_($I_{ph}(f)$/$I_{ph,0})$. The −3 dB bandwidth was calculated from the frequency where the normalized response dropped to −3 dB, equivalent to $I_{ph}(f)$/$I_{ph,0}$ = 0.707. Interpolation between the two frequencies closest to −3 dB was applied according to $f_{-3dB}=\;f_{1}+\left(\frac{-3-R_{1}}{R_{2}-R_{1}}\right)\times \left(f_{2}-f_{1}\right)$, where $R_{1}$ and $R_{2}$ represent the normalized responses in dB at $f_{1}$ and $f_{2}$, respectively. Using this technique, the estimated −3 dB bandwidths were found to be around 716 Hz under white LED illumination and 775 Hz under blue LED illumination. The increased bandwidth under blue illumination shows that carrier generation and collection occur faster under blue excitation, and this is in line with the increased spectral response of the device at the blue end [22]. This shows direct evidence of the modulation response beyond steady-state photo current and ON-OFF switching tests [22].

One may observe that the extracted bandwidths of about 775 Hz for blue illumination and 716 Hz for white illumination indicate the capability of n-Si/p-NbSe_2_ photodiode to detect the modulated visible light signal within the sub-kHz range. This allows us to present stronger comparative evidence with just ON/OFF switching and confirms that the device possesses a noticeable dynamic characteristic during operation under the modulation by an LED. However, the achieved bandwidths are still much smaller than necessary for high-speed VLC links. In addition, the low-speed performance can result from a high resistance value of the investigated photodiode, increasing the RC (C: capacitance) time constant and causing trap-assisted charge carriers’ movement near the heterojunction boundary. The capacitance characteristic, the small-signal AC characteristic, and the eye diagram were not measured in the current experiment, and the investigated device should be considered as one of the candidates for low-frequency VLC applications, but not yet for high-speed VLC receivers.

The recent literature shows that NbSe_2_ is not usually the main light-absorbing layer, but it strongly improves photodetection when it forms a junction by facilitating carrier separation and charge transport. A study concerned with NbSe_2_/Nb_2_O_5_ heterostructure reported stable UV-visible photoresponse, confirming that NbSe_2_-based interfaces can enhance photosensitivity under illumination [2], while another study, which considered graphene/WSe_2_/NbSe_2_ van der Waals photodetector, reached a responsivity of 0.287 AW^−1^ with EQE% = 88%, showing the beneficial role of NbSe_2_ as an efficient carrier-collection layer [3]. In agreement with these reports, our SNS (n-Si/NbSe_2_) photodiode also exhibits clear light sensitivity, with figures of merit listed in Table 2, and compared with the literature in Table 3, indicates that the n-Si/p-NbSe_2_ junction effectively supports photocarrier generation and extraction. Thus, our results are consistent with the literature trend that NbSe_2_ enhances photoresponse mainly through heterojunction-assisted transport rather than as a standalone absorber.

As an important addition, we recall that recent studies have further emphasized that the performance of nanoscale heterostructure devices is strongly governed by interface quality, charge transport pathways, defect/trap states, and material/process stability. For example, Dastgeer et al. demonstrated that atomically engineered van der Waals heterostructures can enable high-speed non-volatile memory operation and multibit storage by controlling interfacial transport and charge storage processes [42]. In addition, recent progress in high-efficiency perovskite solar cells has shown that strong light absorption, efficient charge extraction, defect passivation, interfacial engineering, and scalable fabrication are key factors for improving optoelectronic device performance and stability [43]. These reports support the broader view that carefully designed junction interfaces and controlled carrier transport are essential for developing efficient, stable, and scalable optoelectronic devices. Accordingly, the present n-Si/p-NbSe_2_ heterojunction is investigated as a simple Si-compatible structure in which interfacial carrier separation and visible-light absorption can be exploited for photodetection and potential VLC-related applications.

## 4. Conclusions

In this work, the n-Si/NbSe_2_ heterojunction fabricated by the thermal evaporation technique under low vacuum pressure showed a clear response to different light sources, which confirms the successful formation of an active photodiode structure. The device performance changed with wavelengths, indicating that the junction is sensitive to both visible and near-infrared illumination. Among the tested conditions, the strongest overall performance was obtained under blue. IR illumination also produced notable responsivity. In Addition, the modulation-response analysis confirmed that the n-Si/p-NbSe_2_ photodiode can follow blue and white LED signals in the sub-kHz range, although full VLC data transmission experiments are still required to validate complete communication-link performance. These results show that the deposited NbSe_2_ layer plays an important role in improving carrier separation and charge transport at the interface with n-Si. Generally, the study demonstrates that the thermal evaporation technique can be used to prepare a functional and low-cost heterojunction photodiode with promising optoelectronic behavior. The obtained responsivity, quantum efficiency, detectivity, and noise characteristics suggest that the device can operate effectively as a photosensor over a broad spectral range. Therefore, the SNS heterojunction can be considered a promising candidate for future visible light communication, optical sensing, and broadband photodetection applications.

## Figures and Tables

**Table 1:** The SNS photodiode characteristics measured at 300 K in the dark and under light.

	Richardson–Schottky				Chueng’s Function					
Light	$n_{F}$	$n_{R}$	${q\phi }_{b-F}$ (eV)	${q\phi }_{bR}$ (eV)	$n_{F}$	$n_{R}$	${q\phi }_{bF}$ (eV)	${q\phi }_{bR}$ (eV)	$R_{sF}$ (MΩ)	$R_{sR}$ (MΩ)
Dark	4.51	3.38	0.79	0.81	2.19	1.90	0.87	0.89	2.76	7.72
White	4.39	3.59	0.80	0.81	1.64	5.14	0.84	0.85	3.28	0.90
Red	4.93	5.03	0.78	0.78	1.58	6.33	0.78	0.85	3.52	0.42
Tungsten	6.05	4.58	0.74	0.76	2.02	1.24	1.00	0.70	2.93	1.15
Blue	4.17	2.45	0.77	0.79	2.48	1.50	0.64	0.56	1.35	0.27
Infrared	4.35	5.34	0.75	0.75	2.83	1.42	0.54	0.68	0.62	0.55

Subscripts F and R are forward and reverse biased conditions, respectively.

**Table 2:** Figures of merit for SNS photodiodes recorded for various light sources of different wavelengths. Data is recorded for the highest LED optical power.

Condition	*R* (AW^−1^)	EQE%	NEP (×$10^{-14}\;WHz^{-1/2}$)	D* (10^12^ Jones)
Blue	3.79	1160	4.43	3.91
White	3.68	809	4.57	3.79
Red	0.86	169	19.5	0.89
Tungsten	0.80	182	21.0	0.83
IR	1.27	161	13.20	1.31

NEP and D* were estimated at −5 V by including both shot-noise and Johnson–Nyquist-noise contributions; low-frequency 1/f noise was not experimentally quantified.

**Table 3:** Figures of merit for photodiodes used in VLC and IR technology.

Photodiode/Device	Responsivity (AW^−1^)	EQE (%)	NEP (W/Hz^1/2^)	D* (Jones)	Reference
n-Si/p-SeO_2_/SiO_2_	0.12	15	-	1.30 $\times \;10^{10}$	[33]
polyoxometalate/p-Si	1.07	125	3.7 $\times \;10^{-11}$	2.1 $\times \;10^{9}$	[35]
Mg_2_Si/Si	0.44	88.98	6.38 $\times \;10^{-12}$	1.56 $\times \;10^{11}$	[36]
Nb_2_O_5_/n-Si	0.44	95.36	3.89 $\times \;10^{-13}$	1.25 $\times \;10^{10}$	[37]
Lanthanide doped nanoparticles	0.51	64.9	-	6.9 $\times \;10^{9}$	[38]
n-Si/p-NbSe_2_ (White)@$V_{R}=6.0\;V$ $P_{Opt}=$50 μW	3.68	809	4.57 $\times \;10^{-14}$	3.79 $\times \;10^{12}$	This work

## Data Availability

The data that support the findings of this study are available from the corresponding author upon reasonable request.

## References

[1] Gupta S., Roy D., Bose S., Dixit V., Kumar A. (2024). Illuminating the future: A comprehensive review of visible light communication applications. Opt. Laser Technol..

[2] Xu X., Lu C., Wang Y., Bai X., Liu Z., Zhang Y., Hua D. (2023). Two dimensional NbSe_2_/Nb_2_O_5_ metal–semiconductor heterostructure-based photoelectrochemical photodetector with fast response and high flexibility. Nanoscale Horiz..

[3] He S., Yin C., Zhang L., Chen Y., Peng H., Shan A., Zhao L., Gao L. (2025). All-2D asymmetric self-powered photodetectors with ultra-fast photoresponse based on Gr/WSe_2_/NbSe_2_ van der Waals heterostructure. J. Mater. Sci. Technol..

[4] Su C., Li M., Yan H., Zhang Y., Li H., Fan W., Bai W., Liu X., Wang Q., Yin S. (2025). PdSe_2_/NbSe_2_ heterojunction photodetector with broadband detection and polarization sensitivity. ACS Appl. Mater. Interfaces.

[5] Wu H., Wang Y., Xu Y., Sivakumar P.K., Pasco C., Filippozzi U., Parkin S.S.P., Zeng Y.-J., McQueen T., Ali M.N. (2022). The field-free Josephson diode in a van der Waals heterostructure. Nature.

[6] Feng S., Li N., Liu K., Li B., Dong C., Wu Q. (2025). A Cross Q-Learning Assisted Resource Allocation for User-Centric Optical Wireless Communication Networks. IEEE Trans. Green Commun. Netw..

[7] Gao M., Xu G., Song Z., Zhang Q., Zhang W. (2025). Performance Analysis of LEO Satellite-Assisted Deep Space Communication Systems. IEEE Trans. Aerosp. Electron. Syst..

[8] Fahad O.A., Ramizy A., AlRawi B.K. (2025). Fabrication and characterization of a visible photodetector based on a germanium/n-type silicon heterojunction using thermal evaporation deposition. J. Opt..

[9] Jain A.K., Malar P. (2023). Fabrication and study of Si/Sb_2_Se_3_ heterojunction-based visible light photodetectors. J. Mater. Sci. Mater. Electron..

[10] Marezio M., Dernier P.D., Menth A., Hull G.W. (1972). The crystal structure of NbSe_2_ at 15 K. J. Solid State Chem..

[11] Ferguson A.J., Farshchi R., Paul P.K., Dippo P., Bailey J., Poplavskyy D., Khanam A., Tuomisto F., Arehart A.R., Kuciauskas D. (2020). Defect-mediated metastability and carrier lifetimes in polycrystalline (Ag, Cu)(In, Ga) Se2 absorber materials. J. Appl. Phys..

[12] Ryu G., Kim S.W., Mizoguchi H., Matsuishi S., Hosono H. (2012). Superconductivity in a PbFCl-type pnictide: NbSiAs. Europhys. Lett..

[13] Qasrawi A.F., Daragme R.B. (2025). Design and Characterization of Se/Nb_2_O_5_ Interfaces as High Infrared-Absorbers and High Frequency Band Filters. Cryst. Res. Technol..

[14] Qasrawi A.F., Abu Al Rob O.H. (2019). Enhancements of light absorbability, optical conductivity, and terahertz cutoff frequency in stacked layers of selenium via Ag nanoslabs sandwiching. Phys. Status Solidi A.

[15] Xiao C., Liu T., Sun L., Chen L. (2026). Emerging tungsten-based materials for rechargeable metal-ion batteries: Progress and perspectives. Chem. Commun..

[16] Amin A., Cagno C., Wang Y., Yan F. (2025). A review of interface engineering in antimony chalcogenide thin film solar cells. Sol. RRL.

[17] Qasrawi A.F. (2025). Enhanced Dielectric Properties, and Optical Conduction of Amorphous Silicon Thin Films via Ag_2_O Coatings. Silicon.

[18] Patel K., Solanki G.K., Patel K.D., Pataniya P., Pathak V.M., Tannarana M., Chauhan P., Patel M. (2019). Flat band potential determination of NbSe2 photoelectrode using Mott-Schottky plot. AIP Conference Proceedings 2115.

[19] Javaid K., Anjum R., Ali A., Mahmood K., Amin N., Al-Buriahi M.S., Katubi K.M., Alrowaili Z.A., Shehzad U., Anwar H. (2025). Band offset engineering to improve electrical transport properties of p-NiO/n-ZnO heterojunction diode. J. Alloys Compd..

[20] Chiu M.-H., Zhang C., Shiu H.-W., Chuu C.-P., Chen C.-H., Chang C.-Y.S., Chen C.-H., Chou M.-Y., Shih C.-K., Li L.-J. (2015). Determination of band alignment in the single-layer MoS2/WSe2 heterojunction. Nat. Commun..

[21] Fregolent M., Boito M., Disarò M., De Santi C., Buffolo M., Canato E., Gallo M., Miccoli C., Rossetto I., Pizzo G. (2024). Negative activation energy of gate reliability in Schottky-gate p-GaN HEMTs: Combined gate leakage current modeling and spectral electroluminescence investigation. IEEE J. Electron Devices Soc..

[22] Sze S.M., Li Y., Ng K.K. (2021). Physics of Semiconductor Devices.

[23] Latreche A. (2019). Combined thermionic emission and tunneling mechanisms for the analysis of the leakage current for Ga_2_O_3_ Schottky barrier diodes. SN Appl. Sci..

[24] Wei Y., Lan C., Zhou S., Li C. (2023). Recent advances in photodetectors based on two-dimensional material/Si heterojunctions. Appl. Sci..

[25] Zhang B., Tao Y., Kumar Chamoli S., Chen Q., Zhao K., Yu Y., Wang B. (2022). Dynamically control selective photo response in the visible light using phase change material. Opt. Laser Technol..

[26] Zanoon T., Qasrawi A.F., Alawneh I., Khanfar H.K. (2026). MoO_3_ Doping Effects on the Performance of CuO High-Temperature Current Rectifiers. Arab. J. Sci. Eng..

[27] Tan X. (2025). Device Physics of Single-Layer Organic Light-Emitting Diodes with Wide Band Gap Emitters. Ph.D. Thesis.

[28] Myers G.E., Montet G.L. (1970). Optical properties of single crystals of NbSe2 and Nb1. 04Se2. J. Appl. Phys..

[29] Rodrigues I.S.C., Ximenes L.R., Arthur R., Perez-Jimenez R. (2025). Color-selective channel modeling for visible light communication (VLC). IEEE Access.

[30] Ming M.M., Nan Q., Sun L., Chen Y., Xu K., Zhang Y., Liu M., Du S., Liu K., Feng Y. (2021). Lightweight 3D-TiO_2_ nanotube arrays on Ti mesh for promoted photoelectrochemical water splitting. J. Nanoelectron. Optoelectron..

[31] Zhu Q., Yu L., Wen B., Cheng J., Deng Y., Li J. (2024). Passive Q-switched fiber laser based on niSe2 saturable absorber. Opt. Quantum Electron..

[32] Bai H., Guo R., Zhou Y., Feng C., Chen Y., Zhang S., Feng Y., Liu W., Liu K., Guo F. (2026). A room-temperature wireless NO_2_ gas sensor enabled by WO_3_ modified V_2_CT_x_ nanosheets. Chem. Eng. J..

[33] Alharbi S.R.N., Qasrawi A.F., Algarni S.E. (2023). High-performance n–Si/p–SeO_2_/p–SiO_2_ heterojunction photodetectors for potential application in visible light communication technology. Appl. Phys. A.

[34] Donati S. (2021). Photodetectors: Devices, Circuits and Applications.

[35] Shekhawat K., Prajapat P., Gupta G., Negi D., Shyam R., Gupta M., Nelamarri S.R. (2024). Investigation of Ge/Sn/Al_2_O_3_ multilayer structure for photodetector application. Opt. Mater..

[36] Yu H., Ji S., Luo X., Xie Q. (2021). Technology CAD (TCAD) simulations of Mg2Si/Si heterojunction photodetector based on the thickness effect. Sensors.

[37] Hussaini A.A., Esra Yıldız D., Akyildiz O., Bağcı C., Yıldırım M. (2026). High-Performance Broadband Nb_2_O_5_/n-Si Schottky Photodetector for UV–Vis–NIR Self-Powered Applications. Phys. B Condens. Matter.

[38] Fang G., Ji Y., Xiao Q., Dong X., Wu J., Zou J., Xu Y., Xu W., Dong B. (2022). Plasmonic Au@ Ag-upconversion nanoparticle hybrids for NIR photodetection via an alternating self-assembly method. J. Mater. Chem. C.

[39] Bube R.H. (1992). Photoelectronic Properties of Semiconductors.

[40] Andreeva O.N., Braude I.S., Mamalui A.A. (2012). Selenium vacancies and their effect on the fine structure of NbSe2 quasi-two-dimensional single crystals. Phys. Met. Metallogr..

[41] Kelly R., Lima T., Barreto L., Karapetrov G. (2025). Electron and photon induced selenium migration in single crystal 2H-NbSe2. Appl. Phys. Lett..

[42] Dastgeer G., Nisar S., Rasheed A., Akbar K., Chavan V.D., Kim D.-k., Wabaidur S.M., Zulfiqar M.W., Eom J. (2024). Atomically engineered, high-speed non-volatile flash memory device exhibiting multibit data storage operations. Nano Energy.

[43] Dastgeer G., Nisar S., Wajid Zulfiqar M., Eom J., Imran M., Akbar K. (2024). A review on recent progress and challenges in high-efficiency perovskite solar cells. Nano Energy.

